# Cognitive diagnostic analysis of mathematics key competencies based on PISA data

**DOI:** 10.1371/journal.pone.0315539

**Published:** 2025-02-24

**Authors:** Yi Zhang, Baixiang Zhao, Min Jian, Xiaopeng Wu

**Affiliations:** 1 College of Education, Guangzhou University, China; 2 School of Fine Arts and Art Design, Tianshui Normal University, China; 3 College of Life Sciences, Northeast Normal University, Changchun, China; 4 Faculty of Education, Northeast Normal University, Changchun, China; 5 Faculty of Education, The University of Hong Kong, Hong Kong, China; RMIT University, VIET NAM

## Abstract

As a new generation of assessment instrument, cognitive diagnosis integrates the measurement objectives into the cognitive model to diagnose the fine-grained knowledge of students. Taking the PISA 2012 dataset in mathematics from Shanghai, Hong Kong, Macau and Taiwan as the research subject, this study constructed a cognitive model with the attributes of Mathematical Abstraction, Logical Reasoning, Mathematical Modeling, Intuitive Imagination, Mathematical Operation and Data Analysis, and made an analysis of the mastery of students’ mathematical competencies of different attributes in four regions, and the learning paths of the students’ mathematical competencies were constructed. The results showed that Shanghai had the obvious advantages in each attribute; the mastery mode of Hong Kong, Macau and Taiwan showed a common trend, and they all indicated a relatively low percentages of competencies in *Logical Reasoning* and *Intuitive Imagination*. In terms of the learning paths, the learning paths in the four regions reflected diversities, but obvious main learning paths existed. Majority of the knowledge states’ abilities were below 0. While in Hong Kong, Taiwan, and Macau, more knowledge states’ abilities were above 0. This research provided a reference for the systematic analysis of students’ knowledge status and learning path.

## 1. Introduction

### 1.1. The definition and attribute division of mathematical literacy

Over the past two decades, particularly since Denmark’s publication of the KOM report in 2002 (KOM is an acronym for “Competencies and the Learning of Mathematics”), there has been a surge of global interest in mathematical competency across varied contexts and purposes [1]. Recognized as a core component of 21st-century skills, the importance of mathematical competency continues to grow [2]. Contributions from individual researchers, academic groups, various organizations, and national and state-level policy initiatives have driven competency-oriented educational reforms. For instance, the U.S. *National Assessment of Educational Progress* (NAEP) assessment of mathematical competency has undergone over 40 years of development, now serving as a vital tool for understanding the state of mathematics education across U.S. states [3]. Similarly, recent educational guidelines and frameworks in countries like Australia (e.g., the National Curriculum Framework) emphasize mathematical competency as a core aim of school education [4]. In Sweden, competency-based mathematics education began with curriculum reform in 1994 and was further solidified in the 2011 syllabus, where goals are explicitly based on competencies [5]. International assessments like *Program for International Student Assessment* (PISA) and *Trends in Mathematics and Science Study* (TIMSS) also prioritize mathematical competency as a key evaluation target, underscoring its growing significance within global education. A review of literature has highlighted the important role of mathematical competency, but so far there is no agreed definition of mathematical competency.

Mathematical competencies, abilities or literacies are common terms used in defining the concept of mathematical competencies. Since they are all quite close in connotation, this study will not make specific distinctions among these definitions. Kilpatrick et al. consider the mathematical competency as a tool to deal with the non-mathematical problems, which include the ability to master mathematical knowledge, and solve the mathematical and non-mathematical problems with mathematical knowledge [6]. Jablonka proposes that mathematical competency is a kind of practical experience, the concept of which varies under different stakeholder’s value systems [7]. Therefore, two types of value-oriented mathematics have been formed: ability-oriented and application-oriented. These two complement each other in the study of mathematical competency. De Lange states the current understanding toward mathematical competency does not have cultural differences in consideration [8]. Madison and Steen have agreed that people with mathematical competency are not necessarily to be experts in mathematics although no consensus on what they should obtain [9]. In the Danish KOM-project *Competencies and Learning of Mathematics*, the concept of mathematical competency has been expanded. Mathematical competency is regarded as a quality of latent internalization of mathematical knowledge and effective control of one’s own behavior under the premise of basic mathematics knowledge [10]. In 2000, *International Life Skills Survey* in Australia summarizes mathematical competency as a gathering ability for people to deal with mathematical questions in daily life, including skills, knowledge, beliefs, temperament, thinking habits, and communication skills needed in the problem-solving process [11]. In 1999, the *Organization for Economic Cooperation and Development* (OECD) first proposes the definition of mathematics competency in PISA. Mathematical competency is “an individual’s capacity to reason mathematically and to formulate, employ, and interpret mathematics in a variety of real-world contextual environments. It includes concepts, procedures, facts and tools to describe, explain and predict phenomena. The defined concept assists individuals with an understanding of the role that mathematics plays in the world and to make the well-founded judgments and decisions needed by constructive, engaged and reflective 21st century citizens” [12].

According to the concept defined in OECD, studies have also divided the attributes of mathematical competency. The *National Association for Teacher Education* (NCTM) issues *The Outline of Action: Recommendations for School Mathematics* in the 1980s. It is believed that the highly evaluated psychological process of logical reasoning, information processing and decision-making should be taken into consideration in mathematical application. Mathematics teachers and mathematics curriculum should set goals to develop the logic, concept and language of mathematics [13]. Five goals have been put forward by NCTM to cultivate the mathematical competency, which are, to value mathematics, to be confident in mathematical ability, to solve mathematical problems, to communicate mathematically and to reason mathematically [14]. Jablonka summarizes five characteristics of mathematical competency: developing human capital, maintaining cultural identity, pursuing social change, creating environmental awareness and evaluating mathematical application [7]. Kaiser and Sriraman conclude five levels of mathematical competency, which are non-literacy, verbal literacy, functional literacy, conceptual and procedural literacy, and multidimensional literacy through the research of PISA [15]. They believe that difficulties exist in accurately distinguishing these five levels of mathematical competency. In December 2017, *Senior High School Mathematics Curriculum Standard* in China summarizes the existing research results and proposes six core mathematical competencies, namely *Mathematical Abstraction, Logical Reasoning, Intuitive Imagination, Mathematical Modeling, Mathematical Operation* and *Data Analysis*.

### 1.2. Learning path and its definition

The most important factor affecting students’ current learning is their existing knowledge, therefore, it is necessary to accurately position students’ cognitive order of existing knowledge in students’ learning. Simon initially put forward the concept of hypothetical advanced learning, which is, the learning path [16]. The reason to call it hypothetical is because the path towards the expected learning goal at different starting points is unknown in advance. It represents the most likely steps the students will follow to develop from initial mathematical ideas into formal concepts. However, recent studies have found that although the learning path representing students’ cognitive development is not linear, it is also not random [17]. The learning path hypothesis reflects a cognitive sequence formed by group learning awareness during the learning process, illustrating a hierarchical and ordered structure of knowledge states. This path not only demonstrates the transformation of group learning awareness into individual learning behaviors but also emphasizes students’ varied starting points, processes, and mediums in learning. Accordingly, learning paths identify continuous stages that support productive learning, guide progress tracking, and provide a pathway for individualized learning development—enabling students to advance from their initial knowledge base toward specific learning objectives [17–19].

Notably, each student’s learning path has unique characteristics, defined by their attribute profile or knowledge state. Additionally, learning path diagrams provide essential information for teachers to design tailored instructional strategies, including customized learning activities, tasks, materials, communication methods, and assessment approaches. These strategies ensure that students have the opportunity to acquire and master knowledge across all attributes comprehensively [20].The learning path represents the most likely steps that students follow when they develop their original mathematical ideas into formal concepts.

### 1.3. The relationship between learning path and learning progression

In the study of this learning roadmap, the commonly used words are *Learning Progression*, *Learning Trajectory* and *Learning Path*. *Learning Progression* emphasizes the organization of learning goals or learning standards; *Learning Trajectory* emphasizes the targeted concept-oriented and corresponding goal-oriented activities promoted by a series of teaching tasks [21]; while *Learning Path* focuses more on the cognitive development process of student learning content, which is a cognitive sequence of different learning knowledge or skills acquisition. The essence of these three is in general the same [22], which all involve the description of the laws of students’ cognitive development [23], pointing to the more complicated ways of thinking, and focus on the organization of learning content to promote one’s understanding and development of these ideas and concepts over a period of time.

However, from the perspective of cognitive diagnosis assessment, there is a sequential relationship between the learning path and learning progression. Learning path is usually considered as an indispensable prerequisite for obtaining the learning progression. We can get the pattern of each student, that is, the state of knowledge, through cognitive diagnosis assessment; then cluster the same knowledge states into the groups, and finally get the learning progression based on the inclusion relationship between the knowledge states [24]. Under the theory of cognitive diagnosis assessment, the learning progression is a ladder-like knowledge classification system with varied high and low levels. Researchers have begun to use cognitive diagnosis as a quantitative analysis method to provide technical support for the construction of a learning progress evaluation system to deeply evaluate students’ knowledge structure [25–26]. With the support of cognitive diagnosis theory, learning paths provide a basis for building learning progress. By sorting and classifying the ability values in different knowledge states, learning progress can be systematically obtained.

### 1.4. Hypothesis of learning path

Attributes can be referred to as skills, knowledge, or cognitive processes in educational measurement [27]. Xin et al. believes that learning path can be depicted by sorting the attributes through the inclusion relationship of a core concept based on cognitive diagnosis assessment [28]. Xu et al. believe that for all the attributes, the possession of lower-level skills is assumed to be the prerequisite for the possession of higher-level skills [29]. Wu et al. construct the path of learning content in mathematics for Grade 8 students in 10 countries including China and the United States according to the inclusion relationship of attributes (knowledge status), and correspondingly made a comparative analysis on a cross-national level using PISA dataset [24]. Chen et al. use the *Space Rule Model* (RSM) in cognitive diagnosis to construct the learning path of elementary school students’ sense of number, which provide a methodological and practical basis for studying the path of mathematical knowledge and ability using cognitive diagnosis assessment [25].

Overall, there exist three hypotheses of learning path under the cognitive diagnostic modeling [24,30].

a. The acquisition of students’ knowledge and skills is gradual and sequential.
b. The constructed learning paths of knowledge or abilities through CDA is the ones that take full consideration of the individual differences between groups. A specific group has a common educational and cultural background, including the characteristics of textbook compilation, teaching style, curriculum arrangement, study habits and other factors. Therefore, it can be considered that different individuals in the group exhibit the attributes at the different stages of development. These attributes link each other through a certain cognitive order and construct the learning path.
c. It is assumed that the collective ideology reflected in the learning process of students of different ability levels can be regarded as the learning path of students.

To conclude, as a new generation of assessment theory, cognitive diagnosis has the greatest advantage of diagnosing the knowledge on fine-grained points, so as to further understand the students’ mastery mode of knowledge. It applies modern statistical thinking and cognitive theory to the psychological assessment model, integrating test objectives into cognitive process model, and then reflects the psychological and cognitive characteristics of the subjects [31]. Through cognitive diagnosis assessment, students’ learning trajectory can be accurately obtained.

This study utilizes the 2012 mathematical dataset from four regions—Shanghai, Hong Kong, Macau, and Taiwan—to compare students’ mastery of the six core mathematical competencies defined in the *Mathematics Curriculum Standard for Senior High School* in China. Based on this comparison, the study constructs and analyzes the learning paths of students in these regions. The aim of this research is to provide a systematic approach to understanding students’ knowledge states and learning abilities. By examining the regional differences in students’ mastery of mathematical skills, this study seeks to explore the effectiveness of educational practices and the potential factors influencing students’ mathematical competencies. Ultimately, the research aims to deepen the understanding of students’ mathematical abilities across these regions and offer broader insights for future curriculum design and teaching strategies.

## 2. Cognitive model construction

### 2.1. Cognitive attributes

With an integration of modern statistical ideas and cognitive psychology theories into the psychological assessment model, cognitive diagnostic assessments measure students’ specific knowledge structures and processing skills, providing information about their cognitive strengths and weaknesses [32]. As mentioned earlier in the article, Cognitive attributes play a crucial role in cognitive diagnostic assessment. Cognitive attributes are invisible cognitive states hidden behind assessment tasks that are difficult to observe directly [33]. Leighton and Gierl define the attributes in the process of cognitive diagnosis as the processing skills and knowledge structure needed to complete a task [34]. Attributes can be referred to as skills, knowledge, or cognitive processes in educational measurement [27]. It is thus clear that cognitive attribute is a classification based on a certain standard in order to understand students’ knowledge states more precisely [24]. Therefore, being heterogeneous, attributes could be the knowledge, strategy, skill, process, method, etc. necessary to complete the task. It is a description of the internal processing of students’ psychology in the process of solving problem. In this study, the six mathematical competencies defined in *Mathematics Curriculum Standard for Common Senior High School* in China were considered as the cognitive attributes, which were shown in Table 1.

**Table 1:** The division of cognitive attributes of PISA.

Code	Attribute	Definition
MA	Mathematical Abstraction	The thinking process of extracting common and essential attributes or featuresv of a similar mathematical object and discarding other non-essential attributes or features.
LR	Logical Reasoning	It is the process of arriving at specific statements or individual conclusions from general premises through derivation, or deduction
MM	Mathematical Modeling	According to the actual problem to establish mathematical model, mathematical model to solve, and then according to the results to solve the actual problem
II	Intuitive Imagination	The process of perceiving the form and change of things by means of geometric intuition and spatial imagination, and understanding and solving mathematical problems by means of graphics
MO	Mathematical Operation	Mathematical operation refers to the accomplishment of solving mathematical problems according to operation rules on the basis of clear operation object
DA	Data Analysis	By acquiring data, using appropriate methods to sort out, characterize, analyze and infer the data, and finally achieve the understanding of the research object

### 2.2. Q-matrix

The Q matrix is a two-way specification table that links student responses with their cognitive states by indicating the specific attributes in each item [35]. The Q-matrix table provides a straightforward representation of the relationship between items, categories, and attributes [36]. In cognitive diagnosis assessment, Q matrix is the matrix to connect items and their cognitive attributes. 1 in the matrix represents that the corresponding item involves the attribute, while 0 is the opposite. It connects the students’ unobservable cognitive state with the observable answers in the items [36].

PISA administered its first computer-based mathematics literacy assessment as part of its fifth program edition. A total of 32 countries participated in this effort. In this context, 40 min were allocated for the computer-based portion of the test, with math items arranged in 20 min clusters that were assembled with digital reading or problem-solving prompts [37]. Twelve items in PISA 2012 were selected to use for cognitive diagnosis analysis in this study. The main reason to select these twelve was that only these in mathematical tests were publicized so far and jointly tested by the students in the four regions we studied. These items measure key mathematical abilities such as mathematization; reasoning and argumentation; using symbolic, formal, and technical language and operations; and using mathematical tools [38]. According to the six mathematical competencies described before as the attributes of cognitive diagnosis assessment, the attributes of each of the twelve items were calibrated. The six core mathematics competencies, promulgated by the *Ministry of Education of the People’s Republic of China*, have become the core content of the current research. We adopted the expert method during the calibration process. In total, 10 middle school teachers, 2 mathematics teaching and research staff, 13 mathematics education experts and doctoral students have contributed to the calibration process. The attribute with more than 50% proportion in experts’ calibration was determined as selected and was calibrated as 1. The Q matrix was thus formed, as shown in Table 2.

**Table 2:** Q Matrix of 12 items in PISA.

	Item1	Item2	Item3	Item4	Item5	Item6	Item7	Item8	Item9	Item10	Item11	Item12
MA	1	0	0	0	0	0	1	0	0	1	0	0
LR	0	0	0	0	1	0	0	1	1	0	1	1
MM	0	0	0	0	1	0	0	1	0	0	0	1
II	1	0	0	0	0	0	0	0	0	1	1	0
MO	1	1	0	0	0	1	1	1	1	1	1	1
DA	0	0	1	1	1	0	0	0	0	0	0	0

## 3. Method

### 3.1. Sample

In this study, students who completed all 12 items were selected as the research objects. A total of 6,561 students’ responses were analyzed in the four areas, including Shanghai (1763), Hong Kong (1376), Macau (1602) and Taiwan (1820). Data for these studies were obtained between June 2021 and August 2021, this study was approved by the Human Subjects Protection Committee of East China Normal University. Approval No: HR-224-2023.

#### Model selection.

The Cognitive Diagnostic Model (CDM) integrates psychometric methods with cognitive psychology to assess and diagnose students’ internal cognitive processes, offering detailed insights into cognitive understanding [33]. At its core, CDM assumes that a student’s ability to meet the criteria of a given task is determined by their mastery of specific latent skills and knowledge, known as cognitive attributes. By having students engage with a carefully structured series of tasks and analyzing their responses, researchers can infer which attributes each student has mastered and which remain underdeveloped. This approach yields fine-grained insights into individual student proficiency profiles, allowing for the identification of latent subgroups within the broader population. Because cognitive diagnosis assessments vary in their assumptions, parameters, mathematical foundations, and applications based on attribute characteristics, various CDM models have been developed to suit different contexts [39]. Consequently, to ensure valid and reliable cognitive diagnostics, it is essential to select an appropriate CDM, guided by both theoretical considerations and empirical model-data fit [40]. To get a model with a better model fit, this study evaluated the parameters of nine cognitive diagnosis models – DINA, DINO, RRUM, ACDM, LCDM, LLM and Mixed Model. Since LCDM works the same way with G-DINA, GDM works the same with LLM when the item responses are dichotomous, G-DINA and GDM were no longer considered in our comparisons. In the model comparison process, the Deviation measures the discrepancy between the model and reality, with a lower Deviation indicating a better fit. The AIC and BIC were used to assess the goodness of fit of CDM models, with a smaller value indicating a better fit of the data to the model [41]. With the employment of the CDM package in software R, we have compared the results of these parameters across the nine models and the results were shown in Table 3.

**Table 3:** Parameter statistics of different models.

Model	Number of parameters	Deviation	AIC	BIC
DINA	87	75268.59	75442.59	76033.23
DINO	87	75193.84	75367.84	75958.48
RRUM	101	74690.90	74892.90	75578.58
ACDM	101	74702.15	74904.15	75589.83
LLM	127	74587.29	74789.29	75474.97
LCDM	127	74508.48	74762.48	75624.67
Mixed Model	105	74591.49	74801.79	75514.63

As shown in Table 3, the LLM performed best according to BIC, while LCDM had the lowest AIC. Additionally, LCDM displayed the smallest Deviation, indicating the closest fit to the observed data, with both LCDM and LLM fitting better than other models. To further refine the selection, item-level fit was evaluated using RMSEA, with a critical value set at 0.1; values below this threshold indicate a good fit [42]. Table 4 demonstrates that the RMSEA values of the 12 items were all below 0.1 in the LCDM model, surpassing the item-level fit of the LLM model.

**Table 4:** *Residuals of 12 items in PISA for* LLM *and LCDM.*

	Item1	Item2	Item3	Item4	Item5	Item6	Item7	Item8	Item9	Item10	Item11	Item12
LCDM	0.006	0.053	0.022	0.021	0.007	0.032	0.012	0.007	0.006	0.004	0.006	0.012
LLM	0.191	0.124	0.083	0.071	0.241	0.109	0.314	0.284	0.127	0.152	0.324	0.291

Although the LLM performed slightly better on BIC, the LCDM model was ultimately chosen for its superior item-level fit and stronger interpretative power in cognitive attribute diagnosis. The LCDM model accurately diagnoses item response data, aligning well with the study’s aim of achieving detailed diagnostic insights. While this choice may involve a slight compromise on model simplicity, its accuracy is essential within the context of this study.

### 3.2. Validation of instrument

#### 3.2.1. Reliability.

The reliability of cognitive diagnosis assessment can be analyzed from two aspects. First, the reliability of cognitive diagnosis test can be measured by Cronbach’s alpha (*a*) under the Classical Testing Theory (CTT). In our study, Cronbach’s alpha *a* =.77. The second aspect is to calculate the test-retest reliability. Since the probabilities of the attributes remain unchanged for each subject, we can obtain the index through calculating the correlation between the probabilities of the attributes mastered by the same subjects in two successive measurements [43]. The reliabilities of the six attributes in the test were.72,.81,.89,.69,.94 and.83 specifically. All the indicators were all greater than 0.70 with high reliability, except for the reliability of the attribute *Mathematical Operation* (.062), which was slightly lower than the others.

#### 3.2.2 Discrimination.

In Cognitive Diagnosis Assessment, model accuracy and the item quality can be measured through item discrimination [44]. In the test, discrimination $d_{j}$ usually defined as


$$d_{j}=P_{j}\left(1\right)-P_{j}\left(0\right)$$

where $P_{j}\left(1\right)$ fers to the probability of answering the item *j* correctly when mastering all the attributes of the item *j* and $P_{j}\left(0\right)$ fers to the probability of answering the item *j* correctly without mastering all the attributes of it. The smaller $d_{j}$, the smaller the impact of mastering the attribute on the question and the smaller the degree of discrimination. On the contrary, the higher the degree of discrimination.

A higher degree of discrimination is a sign of high-quality test items. In our study, $d_{j}$ was respectively 0.90, 0.64, 0.22, 0.38, 0.38, 0.55, 0.78, 0.77, 0.81, 0.98, 0.27 and 0.84 through LCDM model analysis. Although $d_{j}$ for 2nd, 3rd, 4th and 11th items were 0.22, 0.38, 0.38 and 0.27 respectively, which were lower; the $d_{j}$ of all the other items were all greater than.7, which were all acceptable especially for the 1st, 7th, 8th, 9th, 10th and 12th items.

## 4. Results

### 4.1. Analysis of students’ mathematical competencies in the four regions

LCDM model which had the best model fit was selected to assess the parameters of the data in this study. The Bayesian Expected a Posteriori Estimation (EAP) was employed through the assessment process, the feature of which was to use posterior distribution to summarize data and get inferences. It calculates the posterior mean or median instead of finding a certain extreme value - mode. EAP is simple, efficient and stable, and is a better choice in the ability parameter estimation methods. The mastery of the students’ six mathematical competencies in the four regions was obtained, and its proportion distribution was shown in Table 5.

**Table 5:** The proportion distribution of six core mathematical competencies in four regions.

	Mathematical Abstraction	Logical Reasoning	Mathematical Modeling	Intuitive Imagination	Mathematical Operation	Data Analysis
Shanghai	0.78	0.79	0.71	0.39	0.75	0.90
Hongkong	0.79	0.76	0.47	0.56	0.61	0.87
Macau	0.47	0.69	0.45	0.38	0.57	0.81
Taiwan	0.56	0.66	0.66	0.39	0.54	0.85

**Fig 1:**
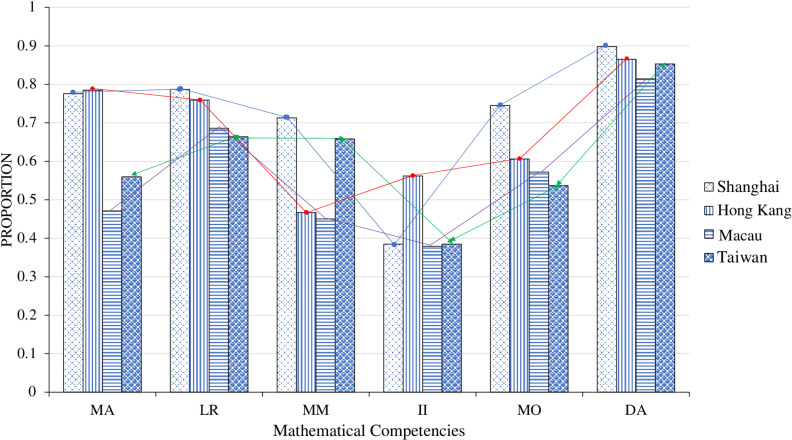
Probability distribution of mastery of students’ six mathematical competencies in four regions.

To reflect the mastery of students’ mathematical competencies in the four regions more intuitively, according to Table 5, the line chart was obtained, which was shown in Fig 1.

According to Fig 1, comparison across different districts, students in Shanghai had obvious advantages in the six mathematical competencies, and the mastery was relatively equal for each competency. Except for *Intuitive Imagination* which had a relatively low percentage of mastery, which was only 38.50%, the mastery probabilities of other attributes were higher than those of the other three regions. The reasons that contributed to the success of Shanghai students’ performance in PISA were, for instance, first, it could benefit from China’s long-term mathematical education tradition and methods, and secondly, it was highly related with the 30 years’ experience of basic education in Shanghai and the reform of mathematics curriculum and teaching [45]. Under the premise of *Reform and Opening Up*, Shanghai continues to explore the reform of mathematics education, and has accumulated many experiences including mathematics variant exercises, small steps forward teaching, emphasizing the activities of the teaching and research group, insisting on teacher training to promote the professional development of teachers, and promote the development of every student. The key competencies of Hong Kong students were second only to those of Shanghai. Except for the attributes of *Mathematical Modeling*, all other attributes were better than those of Macau and Taiwan, and the attributes of *Mathematical Abstraction* were even better than those of Shanghai. Notably, Hong Kong students exhibited high performance in *Intuitive Imagination*, which may be related to education policies that emphasize creativity and flexibility. For example, Hong Kong’s “Learning to Learn” curriculum reform encourages critical thinking and creativity across disciplines, which includes STEM subjects where intuition and imagination are especially valued [46]. The cultural context in Hong Kong also places high importance on adaptability and innovation, potentially fostering an educational environment where students feel more comfortable exploring intuitive approaches [47]. This focus on adaptability aligns with Hong Kong’s internationalized education system, which integrates elements from various global education practices and promotes an open-minded, explorative approach.

The mediocre performance of Macau students in various mathematical abilities, particularly their weak *Mathematical Abstraction* skills, can largely be attributed to the prevalent practice of grade retention. Research by Sit et al. indicates that retained students in Macau significantly lag their non-retained peers in the PISA 2012 mathematics literacy assessment [48]. Although grade retention policies are intended to address academic deficits, their actual effectiveness is limited. Retained students, lacking systematic academic support, fail to master advanced mathematical concepts comprehensively, especially in developing abstract reasoning skills. Moreover, the low self-confidence and high anxiety induced by retention further undermine their mathematical understanding, resulting in overall academic stagnation.

Taiwanese students’ strong performance in *Data Analysis* and *Mathematical Modeling* can be attributed in part to the structural adjustments made under the 12-year Basic Education reform. This reform introduced interdisciplinary courses and context-based learning, exposing students to real-world problem-solving tasks that enhance skills in data processing, logical reasoning, and model construction. Emphasizing the principle of “connecting mathematics with life,” the curriculum employs inquiry-based, context-rich tasks that foster students’ data-handling and modeling competencies [49]. However, a focus on structured analytical skills and written problem-solving has limited the development of intuitive manipulation and spatial imagination abilities [50]. Additionally, while textbooks emphasize high cognitive demand tasks, the exam-driven nature of the education system often deprioritizes hands-on, exploratory learning, resulting in comparatively weaker intuitive and spatial skills among students. Overall, students had the best grasp of the attributes of *Data Analysis*, however, they had the worst grasp of the attributes of *Intuitive Imagination*. Therefore, there is a necessity to strengthen the content design in curriculum reform and teaching design, and to cultivate students’ intuitive imagination ability.

### 4.2. Learning paths of students’ mathematical competencies in the four regions

The premise for the establishment of the learning path is that the students’ learning process has levels of relationship, that is, attributes at a low level should be easy to master, and attributes at a high level should be more difficult to master. According to this hypothesis, the knowledge states obtained by the assessment were clustered, and based on the inclusion relationship between the knowledge states, diagrams of the learning path of students from the four regions of China have been drawn. Students with different knowledge states can choose different learning paths in the following path diagrams.

**Fig 2:**
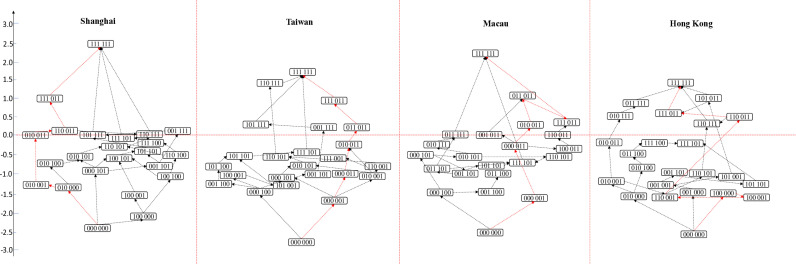
Students’ mathematics learning path in Shanghai, Taiwan, Macau and Hong Kong.

In this study, we applied the package mirt in R to calculate the ability value of each student using the 3PL parameter model, and the ability value of the knowledge state class was obtained by averaging the same class of the knowledge states of the students. In the construction of the learning path, the learning path is constructed based on the inclusion relationship between attributes. As shown in Fig 2 of Taiwan, compared with (011011), subjects belonging to knowledge state (111011) have mastered all attributes belonging to knowledge state (011011), and have mastered other additional attributes, which can be recorded as (011011) ⊂  (111011). We then say there is a hierarchical relationship between the two kinds of knowledge states, that is, there is a path of (011011) →  (111011). In Fig 2, it is (000000) ⊂  (000001) ⊂  (000011) ⊂  (010011) ⊂  (011011) ⊂  (111011) ⊂  (111111). Therefore, there is a learning path of (000000) →  (000001) →  (000011) →  (010011) →  (011011) →  (111011) →  (111111).

The five digits in the box represent the knowledge status. The knowledge statuses connected by red arrows represent the main learning paths.

As shown in Fig 2, Shanghai students’ learning paths in mathematics exhibit significant diversity and a hierarchical structure, with a primary learning path: (000000) →  (010000)  →  (010001)  →  (010011)  →  (110011)  →  (111011)  →  (111111). This pathway reflects a typical cognitive development sequence for many Shanghai students: they first acquire *Logical Reasoning*, then gradually master *Data Analysis*, *Mathematical Operation*, *Mathematical Abstraction*, and *Mathematical Modeling*, and finally, *Intuitive Imagination*. This sequence suggests that *Logical Reasoning* poses relatively little challenge for Shanghai students, whereas *Intuitive Imagination* is the most difficult to master, aligning with previous findings that *Logical Reasoning* has the highest probability of mastery among the four regions, while *Intuitive Imagination* is the least mastered attribute. The diversity of Shanghai students’ learning paths provides direction for personalized instruction. Teachers could, for example, design learning plans based on students’ current knowledge states, supporting those who have acquired *Logical Reasoning* but not yet *Mathematical Operation* by introducing *Data Analysis* and *Mathematical Operation* in applied contexts to foster steady progress. For students struggling with *Intuitive Imagination*, spatial thinking and geometric intuition training could be introduced, specifically through tasks that build spatial visualization skills.

In Taiwan, as shown in Fig 2, the main learning path in mathematics key competencies mastery follows (000000)  →  (000001)  →  (000011)  →  (010011)  →  (011011)  →  (111011)  →  (111111). Most students initially develop *Data Analysis* skills, then progressively acquire other attributes like *Mathematical Operation* and *Logical Reasoning*, with *Intuitive Imagination* typically mastered last. This pattern is closely related to Taiwan’s foundational educational reforms, where *Data Analysis*, a broadly applicable basic skill, is prioritized early in learning, while *Intuitive Imagination*, requiring spatial imagination and geometric intuition, remains underemphasized in early instruction. Although students show high competency in certain knowledge states, many students’ ability values are between -0.5 and -1.5, indicating that lower-ability students have a wider distribution of knowledge states and a more scattered cognitive process. Teachers can address this by structuring the instructional sequence to align with the cognitive order of the main learning path, starting with a focus on foundational skills like *Data Analysis*, and then guiding students through more advanced attributes like *Mathematical Operation* and *Logical Reasoning*. This progressive, path-based approach can help students develop steadily across key knowledge points, preventing cognitive gaps or delays.

In Fig 2, the learning paths of Macau students were more complex in the low-level stage, showing the interweaving state of multipaths, which means that students in the low-level stage had different learning strategies. The learning paths were relatively clear in the high-level stage. There were relatively few classes of knowledge status with ability values greater than 0, indicating that the learning process of students in the high-level stage was basically the same. The primary learning path for Macau students mirrors that of Taiwanese students: (000000)  →  (000001)  →  (000011)  →  (010011)  →  (011011)  →  (111011)  →  (111111). However, Macau students display higher ability values in key knowledge states, such as (000011) (010011) (011011), and (111111), suggesting greater proficiency and skill at these stages. This indicates that Macau students may have received more targeted training or support in mastering these knowledge stages.

Fig 2 showed the knowledge states of all the students participating PISA in Hong Kong. Through comparison, Hong Kong students had more learning status classes (7 in total) with ability values greater than 0. This shows that students had a diversified state of learning in the high-level stage. But overall, students in Hong Kong had relatively low abilities. However, the overall ability values remain lower, with students mastering all attributes (111111) having an ability value of only 1.46, lower than students in the same state in other regions. This suggests challenges in comprehensively mastering all mathematical attributes. The main learning path for Hong Kong students is (000000) →  (100000) →  (100001)  →  (110001)  →  (110011)  →  (111011)  →  (111111), showing a different mastery sequence, with students tending to first acquire *Mathematical Abstraction* before other attributes. This sequence aligns with the “Learning to Learn” curriculum reform in Hong Kong, which emphasizes developing abstract reasoning by strengthening *Mathematical Abstraction* in early instruction. Thus, Hong Kong’s math teaching could build on this foundation, guiding students to subsequently master higher-order skills like *Mathematical Operation* and *Logical Reasoning* for balanced development.

In general, students in Shanghai, Macau, Taiwan, and Hong Kong exhibit distinct learning paths characterized by cognitive order and hierarchical structure. The learning paths in Shanghai and Macau were more complicated. It not only directly relates with the cognitive order of students, but also was affected by other factors such as curriculum arrangement and supplementary tutoring [51]. The learning path of Hong Kong students was the simplest, which may be the result of most students learn according to the curriculum plan. Comparison across the students from all the four regions, the number of the students at the top of the learning path was less, especially those who mastered all attributes in the knowledge state (111111). It also demonstrated the advantages of Shanghai students in mathematics learning. Students in different knowledge states can adapt to themselves and choose different learning paths, which reflects the diversified options of learning. Learning path at different levels represents different ability levels. It clearly describes the development process of students and specifies the clear path and direction for students to develop from the low-level to the high-level learning abilities. Therefore, learning path can not only provide students with personalized and refined diagnosis report, but also a basis for teachers’ remedial teaching. In Shanghai and Macao, differentiated instruction can support students’ steady progression across critical knowledge nodes, while in Hong Kong, reinforcing *Mathematical Abstraction* while broadening skills across other attributes may foster balanced development. The diversity and hierarchy in these learning paths provide essential guidance for personalized instruction, helping educators design suitable strategies to foster continual improvement along students’ respective learning trajectories.

## 5. Conclusion and discussion

In this study, cognitive diagnostic assessment was applied to analyze PISA 2012 data on mathematical competencies among students from Shanghai, Hong Kong, Macau, and Taiwan. By examining the levels of mastery in six core mathematical competencies and constructing learning paths, this research provides a refined view of students’ knowledge states and competency development [24]. The findings indicate that Shanghai students demonstrated notable mastery across competencies, likely due to sustained educational reforms, instructional support systems, and the emphasis on math education within the region’s academic culture. In contrast, students in Hong Kong, Macau, and Taiwan exhibited varied levels of mastery, with strengths in specific competencies but room for improvement in areas like Intuitive Imagination and Logical Reasoning.

The regional differences observed provide meaningful educational implications. Shanghai’s educational model—characterized by targeted teacher training, structured exercises, and iterative curriculum reforms—appears to have been effective in promoting comprehensive competency mastery. Implementing similar strategies in other regions may enhance overall competency levels, especially in critical areas where mastery was lower. Additionally, the varied competency profiles across regions highlight a need for curriculum and instructional adjustments tailored to each region’s specific strengths and weaknesses. For example, addressing gaps in intuitive imagination and logical reasoning may bolster overall competency levels in regions where these areas showed less mastery.

The learning paths constructed from students’ knowledge states offer a valuable framework for teachers seeking to personalize instruction and support students’ progression from foundational to advanced competencies. These pathways enable educators to address learning gaps with targeted, competency-focused strategies. For policymakers, these findings suggest that adaptive educational strategies, which consider the unique regional characteristics and prioritize relevant competencies, could lead to improved mathematical competency across diverse educational contexts. By aligning curriculum and teaching methods more closely with students’ specific learning needs, educational systems can foster deeper engagement and competency development.

In sum, this study emphasizes the importance of adaptable and structured educational strategies. Shanghai’s experience underscores the positive impact of consistent educational support on competency development, suggesting that similarly structured educational reforms could benefit other regions. The variation in regional mastery levels calls for curriculum adjustments aimed at addressing specific competency gaps, such as those in logical reasoning and intuitive imagination. Through adaptive policies and a deeper understanding of learning paths, educators and policymakers can better tailor teach practices, ultimately enhancing student outcomes in mathematics education.

## 6. Limitation

All research inevitably has its limitations. This study relies solely on data from the PISA 2012 cycle, as item-level data from other years remains unavailable, restricting both the time span and potential for longitudinal analysis. Although PISA conducts assessments every three years, each cycle samples different groups of 15-year-old students, making it impossible to track the progress of individual students over time. Recently, scholars have explored longitudinal cognitive diagnostic theories [18,52,53], proposing that longitudinal data collection within this framework could better reveal learning trajectories and educational patterns. Future research could address these limitations by applying a longitudinal cognitive diagnostic approach and, where feasible, integrating data across multiple PISA cycles. This approach would extend the time span of the data, allow for cross-validation of findings, and enable more nuanced exploration of developmental trends across years.

Secondly, as a data-driven study, the findings are influenced by the framework and constraints inherent in the PISA assessment itself, rather than reflecting the realities of student learning across diverse educational contexts. This reliance on standardized assessment data means that the study may not fully capture the complexity of students’ mathematical competency, which is shaped by a wide range of factors beyond the test items. Furthermore, the interpretation of cognitive attributes based solely on PISA data may be incomplete or overly reductionist. To mitigate this limitation, future studies could combine PISA data with other qualitative methods such as interviews, classroom observations, or surveys, which would provide deeper insights into how students’ mathematical abilities manifest in real-life learning environments and how they may evolve over time.

Another notable limitation is the subjectivity involved in defining cognitive diagnostic attributes. The classification of these attributes, while informed by theoretical considerations, could introduce bias that affects the results. For instance, different researchers might categorize certain competencies in various ways, leading to inconsistencies in interpretation. To address this, future research could incorporate more robust validation procedures, such as expanding the scope of experts or pilot studies, to refine the cognitive attributes and ensure greater consistency and objectivity in their definition.

## Supporting information

S1 File All analytical data. (ZIP)s
